# Cholesterol metabolism pathways – are the intermediates more important than the products?

**DOI:** 10.1111/febs.15727

**Published:** 2021-02-17

**Authors:** Yuqin Wang, Eylan Yutuc, William J. Griffiths

**Affiliations:** ^1^ Swansea University Medical School UK

**Keywords:** cholestenoic acids, COVID‐19, G protein‐coupled receptors, glutamate receptors, inborn errors of metabolism, mass spectrometry, nuclear receptors, oxysterols, sterols

## Abstract

Every cell in vertebrates possesses the machinery to synthesise cholesterol and to metabolise it. The major route of cholesterol metabolism is conversion to bile acids. Bile acids themselves are interesting molecules being ligands to nuclear and G protein‐coupled receptors, but perhaps the intermediates in the bile acid biosynthesis pathways are even more interesting and equally important. Here, we discuss the biological activity of the different intermediates generated in the various bile acid biosynthesis pathways. We put forward the hypothesis that the acidic pathway of bile acid biosynthesis has primary evolved to generate signalling molecules and its utilisation by hepatocytes provides an added bonus of producing bile acids to aid absorption of lipids in the intestine.

## Graphical abstract

Have bile acid biosynthesis pathways evolved primarily to generate signalling molecules? and is bile acid formation in the hepatocyte is just a bonus? Intermediates in the conversion of cholesterol to bile acids have important roles in development, immunity, regulation of metabolism and neural homeostasis.

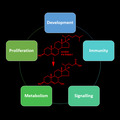

**Abbreviations**
22R‐HC: 22R‐hydroxycholesterol, cholest‐5‐ene‐3β,22R‐diol
24‐HC: 24‐hydroxycholesterol, cholest‐5‐ene‐3β,24‐diol
24S,25‐EC: 24S,25‐epoxycholesterol, 3β‐hydroxycholest‐5‐en‐24S,25‐epoxide
25H,7O‐C: 25‐hydroxy‐7‐oxocholesterol, 3β,25‐dihydroxycholest‐5‐en‐7‐one
25‐HC: 25‐hydroxycholesterol, cholest‐5‐ene‐3β,25‐diol
26‐HC: (25R)26‐hydroxycholesterol, cholest‐5‐ene‐3β,(25R)26‐diol also known as 27‐hydroxycholesterol, 27‐HC
3β,5α‐diHC‐6O: oncosterone, 3β,5α‐dihydroxycholestan‐6‐one
3β,7α‐diHCA: 3β,7α‐dihydroxycholest‐5‐en‐(25R)26‐oic acid
3β,7β‐diHCA: 3β,7β‐dihydroxycholest‐5‐en‐(25R)26‐oic acid
3βH,7O‐CA: 3β‐hydroxy‐7‐oxocholest‐5‐en‐(25R)26‐oic acid
3β‐HCA: 3β‐hydroxycholest‐5‐en‐(25R)26‐oic acid
5,6‐EC: 5,6‐epoxycholesterol, cholestan‐5,6‐epoxide
7‐DHC: 7‐dehydrocholesterol, cholesta‐5,7‐dien‐3β‐ol
7‐OC: 7‐oxocholesterol, 3β‐hydroxycholest‐5‐en‐7‐one, also known as 7‐ketocholesterol
7α,25‐diH,3O‐CA: 7α,25‐dihydroxy‐3‐oxocholest‐4‐en‐26‐oic acid
7α,25‐diHC: 7α,25‐dihydroxycholesterol, cholest‐5‐ene‐3β,7α,25‐triol
7α,25‐diHCO: 7α,25‐dihydroxycholest‐4‐en‐3‐one
7α,26‐diHC: 7α,(25R)26‐dihydroxycholesterol, cholest‐5‐ene‐3β,7α,(25R)26‐triol
7αH,3O‐CA: 7α‐hydroxy‐3‐oxocholest‐4‐en‐(25R)26‐oic acid
7α‐HC: 7α‐hydroxycholesterol, cholest‐5‐ene‐3β,7α‐diol
7β,25‐diHC: 7β,25‐dihydroxycholesterol, cholest‐5‐ene‐3β,7β,25‐triol
7β‐HC: 7β‐hydroxycholesterol, cholest‐5‐ene‐3β,7β‐diol
ABCA1: ATP‐binding cassette subfamily A member 1
ABCG1: ATP‐binding cassette subfamily G member 1
AD: Alzheimer’s disease
AIM2: absent in melanoma 2
APOE: apolipoprotein E
CH25H: cholesterol 25‐hydroxylase, EC:1.14.99.38
ChEH: cholesterol epoxide hydrolase, EC:3.3.2.11
COVID‐19: SARS‐CoV‐2
CRD: cysteine‐rich domain
CSF: cerebrospinal fluid
CYP: cytochrome P450
CYP11A1: cytochrome P450 family 11 subfamily A member 1, EC:1.14.15.6
CYP27A1: cytochrome P450 family 27 subfamily A member 1, EC:1.14.15.15
CYP39A1: cytochrome P450 family 39 subfamily A member 1, EC:1.14.14.26
CYP3A11: cytochrome P450 family 3 subfamily A member 11, EC:1.14.14
CYP3A4: cytochrome P450 family 3 subfamily A member 4, EC:1.14.14
CYP46A1: cytochrome P450 family 46 subfamily A member 1, EC:1.14.14.25
CYP7A1: cytochrome P450 family 7 subfamily A member 1, EC:1.14.14.23
CYP7B1: cytochrome P450 family 7 subfamily B member 1, EC:1.14.14.29
D8D7I: 3β‐hydroxysterol‐Δ8‐Δ7‐isomerase, EC:5.3.3.5
DDA: dendrogenin A
DDB: dendrogenin B
DHCR24: 24‐dehydrocholesterol reductase, EC:1.3.1.72
DHCR7: 7‐dehydrocholesterol reductase, EC:1.3.1.21
EBI2: Epstein–Barr virus‐induced gene 2, GPR183
ER(+)BC: ER‐positive breast cancer
ER: oestrogen receptor
GC: gas chromatography
Gli: glioma‐associated oncogene homolog
GPCR: G protein‐coupled receptor
GPR183: G protein‐coupled receptor 183
GR: glucocorticoid receptor
HD: Huntington’s disease
Hh: Hedgehog
HMGCR: 3‐hydroxy‐3‐methylglutaryl‐Coenzyme A reductase, EC:1.1.1.34
HSD: hydroxysteroid dehydrogenase
HSD11B1: hydroxysteroid 11‐beta dehydrogenase 1, EC:1.1.1
HSD11B2: hydroxysteroid 11‐beta dehydrogenase 2, EC:1.1.1
HSD3B7: hydroxy‐delta‐5‐steroid dehydrogenase, 3‐beta‐ and steroid delta‐isomerase 7, EC:1.1.1.181
IFN: interferon
INSIG: insulin‐induced gene
LC: liquid chromatography
LCAT: lecithin–cholesterol acyltransferase, EC:2.3.1.43
LIPA: lysosomal acid lipase, EC:3.1.1.13
LSS: lanosterol synthase, EC 5.4.99.7
LTP: long‐term potentiation
LXR: liver X receptor
MS: mass spectrometry
MSI: mass spectrometry imaging
NMDAR: *N*‐methyl‐D‐aspartate receptor
NPC1: Niemann–Pick C1
NPC2: Niemann–Pick C2
PD: Parkinson’s disease
Ptch1: patched‐1
SARS‐CoV‐2: severe acute respiratory syndrome coronavirus 2
SCAP: SREBP cleavage‐activating protein
SERM: selective oestrogen receptor modulator
SHH: Sonic hedgehog
SLOS: Smith–Lemli–Opitz syndrome
Smo: Smoothened
SMPD1: acid sphingomyelinase, EC:3.1.4.12
SQLE: squalene epoxidase, EC:1.14.14.17
SREBP‐1c: sterol regulatory‐binding protein‐1c
SREBP‐2: sterol regulatory‐binding protein‐2
TLR: Toll‐like receptor

## Introduction

Cholesterol metabolism has been studied for many decades [1, 2, 3]. In mammals, the products of cholesterol metabolism are bile acids, and steroid hormones and their metabolites [4, 5]. While bile acids and steroid hormones are of undoubted importance, in recent years interest has shifted to intermediates in their biosynthesis and to a category of molecules known as oxysterols [6, 7, 8, 9]. Oxysterols can be defined as oxidised forms of cholesterol or of its precursors. They are formed in the first steps of cholesterol metabolism, mostly by cytochrome P450 (CYP) enzymes [3, 4, 10]. They can also be formed via nonenzymatic reactions both *in vivo* and *ex vivo* [11, 12, 13]. Many oxysterols have biological activity being ligands to, for example nuclear receptors, G protein‐coupled receptors (GPCRs) and glutamate receptors [6, 8, 9, 13].

There are many areas of biology in which oxysterols play a role. At the very beginning of life, oxysterols are key molecules in embryonic development acting along with other sterols to transmit the Hedgehog (Hh) signal [14], a key pathway for fate determination of stem cells and progenitor cells. Oxysterols activate this pathway by binding to Smoothened (Smo), a GPCR found at the cell membranes of primary cilia [15]. Oxysterols also appear as key molecules for definition of neural progenitor fate by activating the liver X receptors (LXRs) [16, 17], while cholestenoic acids, downstream metabolites of oxysterols, are important for survival or death of motor neurons [18]. Oxysterols have been also linked to cancer, through overactivation of Hh signalling and via many other mechanisms [9, 15, 19]. While some oxysterols are oncogenic, others appear to be protective against cancer [9]. Perhaps unsurprisingly as oxidised forms of cholesterol, oxysterols are implicated in the atherosclerotic process being found in atherosclerotic plaques [20]. Oxysterols also appear to be involved in the immune response, having either inflammatory or anti‐inflammatory properties [21, 22, 23, 24], and are generated in response to both bacterial infection and viral infection [25, 26, 27]. There is growing evidence that certain oxysterol may inhibit infection by the SARS‐CoV‐2 virus (COVID‐19) [28, 29, 30, 31].

**Fig. 1:**
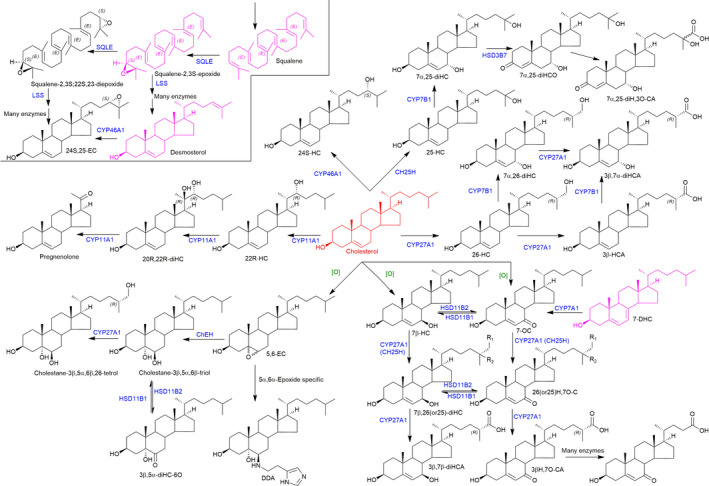
Structure of primary oxysterols and downstream metabolites. Cholesterol is shown in red, cholesterol precursors in purple and oxysterols in black. Enzymes are written in blue, and nonenzymatic oxidation is indicated by [O] in green. In 7β,26‐diHC and 26H,7O‐C, R_1_ = OH and R_2_ = H, while in 7β,25‐diHC and 25H,7O‐C, R_1_ = H and R_2_ = OH.

Accepting that oxysterols are critical biological molecules, it is important to remember that oxysterols are a family of molecules, where small changes in geometry can lead to the difference between activity and inactivity. It is also crucial to be aware that oxysterol concentrations are very often determined following a base hydrolysis step where oxysterols esterified to fatty acids are released, so what is actually being determined is the sum of the free molecules and their esterified versions. Usually, oxysterol esters are more abundant than the nonesterified molecules, but it is the nonesterified molecules that are biologically active. Today, mass spectrometry (MS) in combination with liquid chromatography (LC), that is LC‐MS, or with gas chromatography (GC), that is GC‐MS, is almost exclusively used for oxysterol measurements [32, 33, 34, 35, 36, 37]. In the following sections, we will attempt to summarise the current ‘state of play’ in oxysterol research and endeavour to highlight key unresolved questions. We arrange the review by rotating around the major primary oxysterols derived from cholesterol (Fig. 1), looking at biological activity and downstream metabolites.

## 25‐hydroxycholesterol (25‐HC)

**Fig. 2:**
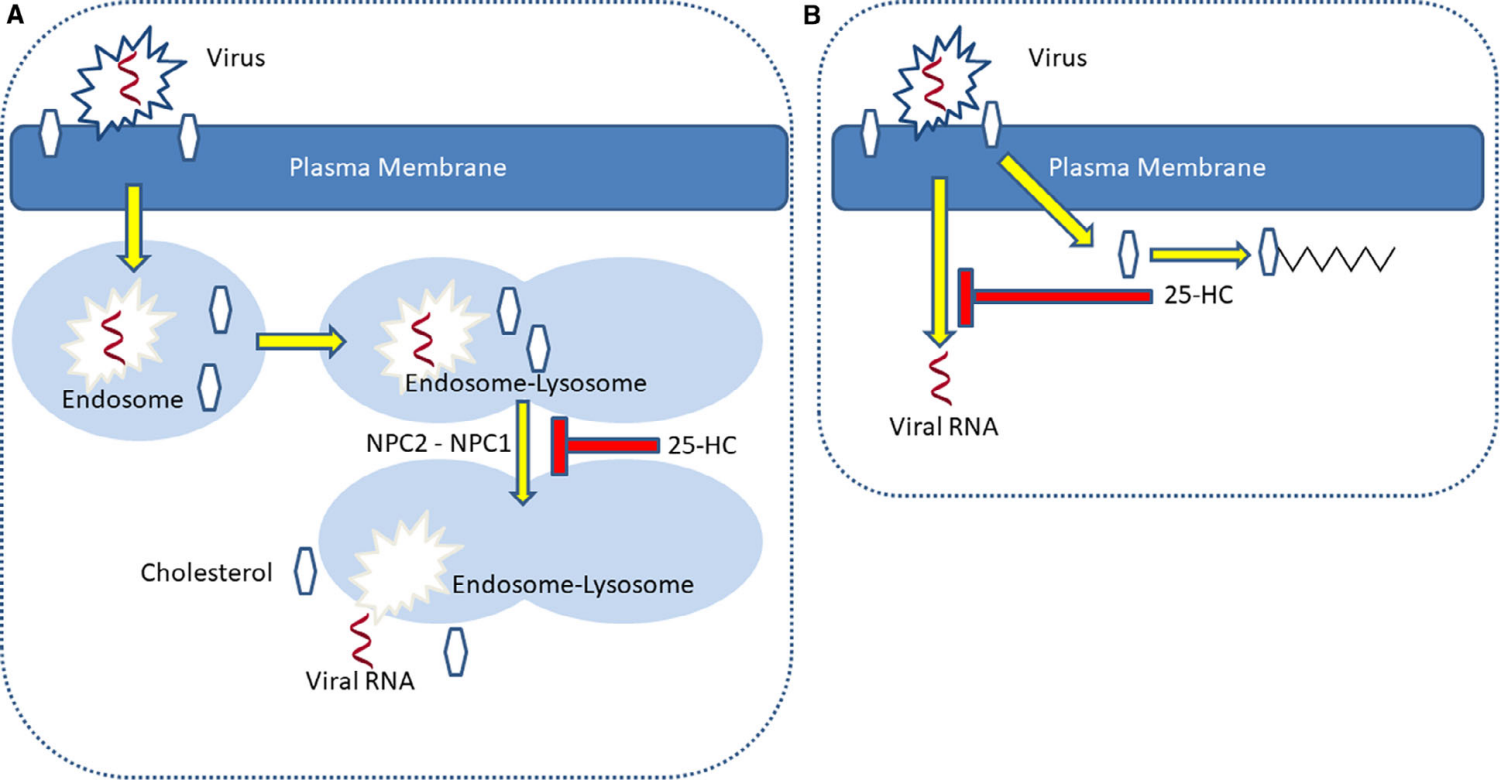
Simplified cartoon representation of the involvement of 25‐HC in protection against SARS‐CoV‐2 infection. (A) The virus enters the cell via endocytosis. Viral RNA escapes from the endosome/lysosome compartment by membrane fusion in concert with NPC2–NPC1‐mediated export of cholesterol. 25‐HC inhibits NPC1‐mediated cholesterol export and traps the virus in the late endosome/lysosome compartment [29]. Alternatively, (B) 25‐HC may activate acyl‐CoA cholesterol acyltransferase and sequester cholesterol as the ester, depleting the availability of plasma membrane nonesterified cholesterol required for membrane fusion and viral entry [31].

25‐HC is an unusual oxysterol in that cholesterol 25‐hydroxylase (CH25H, EC:1.14.99.38) is not a CYP enzyme but is a member of a small group of proteins that utilise a diiron cofactor to catalyse hydroxylation [38]. Note, 25‐HC can also be formed as a minor side product in reactions catalysed by CYP3A4 (EC:1.14.14, CYP3A11 in mouse), CYP27A1 and CYP46A1 [4, 10, 39, 40]. In normal circumstances, the level of 25‐HC is low in tissues and in the circulation [33, 41]; however, upon bacterial or viral infection *CH25H* (*Ch25h* in mouse) is upregulated in activated macrophages with the consequent enhanced formation of 25‐HC [23, 25, 26, 27, 42, 43, 44]. 25‐HC is reported to be anti‐inflammatory and antiviral [21, 26, 27, 45], and this information has stimulated much interest in 25‐HC in relation to SARS‐CoV‐2 [29, 30, 31]. *CH25H* is an interferon (IFN)‐stimulated gene, IFN being induced by Toll‐like receptor (TLR) 3 and TLR4 ligands upon bacterial infection [43, 44]. Upon SARS‐CoV‐2 viral infection, IFN, *CH25H* and other IFN‐stimulated genes are upregulated [29, 31]. Zang *et al*. identified 25‐HC as a potent inhibitor of SARS‐CoV‐2 replication, explaining this by 25‐HC blocking cholesterol export from the late endosome/lysosome compartment and restricting SARS‐CoV‐2 spike protein catalysed membrane fusion [29]. Interestingly, inhibition of Niemann–Pick C1 protein (NPC1), the cholesterol transporter that transports cholesterols out of late endosomes/lysosomes, also inhibited SARS‐CoV‐2 replication, supporting the theory that blocking cholesterol export from late endosomes/lysosomes inhibits viral replication (Fig. 2) [29].

Deficiency in NPC1 (95% of cases) or NPC2 (5% of cases) leads to Niemann–Pick type C disease. While NPC1 protein transports cholesterol across the organelle membrane, NPC2 protein is soluble and carries nonesterified cholesterol to the NPC1 transporter [46]. Niemann–Pick type B disease shows some clinical and biochemically similarities to the type C disease [47, 48], but is genetically different, in that the type B disease results from mutations in the *SMPD1* gene, and deficiency in the enzyme activity of acid sphingomyelinase (EC:3.1.4.12). It has been suggested that acid sphingomyelinase stimulates NPC2‐mediated cholesterol export by converting sphingomyelin to ceramide in the inner membranes of late endosomes [49], and the consequence of its deficiency is enhanced cholesterol content of lysosomes. In support of the hypothesis of Zang *et al*. [29] that blocking cholesterol export from late endosomes/lysosomes inhibits viral replication, Carpinteiro *et al*. [50] have recently found that inhibiting acid sphingomyelinase prevents SARS‐CoV‐2 uptake by epithelial cells, although their explanation for the involvement of acid sphingomyelinase in SARS‐CoV‐2 infection was at the level of ceramide in the outer leaflet of the plasma membrane. An alternative explanation for the antiviral activity of 25‐HC against SARS‐CoV‐2 is provided by Wang *et al*. [31] who found *CH25H* to be induced by SARS‐CoV‐2 *in vitro*, and suggested the antiviral activity of 25‐HC to be via inhibition of membrane fusion through depletion of plasma membrane cholesterol as a consequence of activation of acyl‐CoA cholesterol acyltransferase (EC:2.3.1.26), an enzyme that converts free cholesterol to its cholesteryl ester. Hence, one suggested mechanism of 25‐HC antiviral action is through cholesterol accumulation in the late endosomes, blocking membrane fusion and restricting the virus to this compartment [29], while a second mechanism is through depletion of plasma membrane cholesterol inhibiting viral membrane fusion and entry [31]. A combination of both mechanisms would suggest that 25‐HC can block membrane fusion and viral entry by reducing the available nonesterified cholesterol in membranes by inhibiting transport of cholesterol out of the late endosome/lysosome compartment and through activation of acyl‐CoA cholesterol acyltransferase. In Fig. 2, we present a simplified cartoon representation of the involvement of 25‐HC in preventing viral infection.

It is interesting to note that neither of the antiviral mechanisms discussed above invoked inhibition of SREBP‐2 (sterol regulatory‐binding protein‐2) processing or activation of LXRs, two key regulators of cellular cholesterol status. 25‐HC suppresses cholesterol biosynthesis by binding to the endoplasmic resident protein INSIG (insulin‐induced gene) tethering SREBP‐2 and its escort protein SCAP (SREBP cleavage‐activating protein) within the endoplasmic reticulum and preventing transport of SREBP‐2 to the Golgi for processing to its active form as the master transcription factor for the expression of genes of the cholesterol biosynthesis pathway [51]. Blanc *et al*. [26] proposed this mechanism to partially explain the antiviral action of macrophage produced 25‐HC towards a broad range of viruses, while Dang *et al*. [45] suggested inhibition of SREBP‐2 processing by 25‐HC prevents AIM2 (absent in melanoma 2) inflammasome activation in macrophages and provides an anti‐inflammatory circuit that prevents spurious AIM2 inflammasome activation. 25‐HC is also a ligand to the LXRs [52, 53], LXR activation leads to upregulation of SREBP‐1c and fatty acid synthesis [54], and also of the ABC (ATP‐binding cassette) transporters including ABCA1, ABCG1 and the cholesterol carrier protein apolipoprotein E (APOE) [55, 56]. In combination, the activation of LXR leads to removal of free cholesterol from cells by esterification, transport out of the cell by ABC transporters and its ultimate removal via apolipoproteins in the circulation. Hence, LXR activation by 25‐HC, and also 25‐HC binding to INSIG, may provide additional mechanisms for inhibition of COVID‐19 infection through depletion of membrane cholesterol.

There is now good cell‐based evidence that 25‐HC is protective against SARS‐CoV‐2 through a mechanism involving depletion of membrane cholesterol. The multiple biological activities of 25‐HC suggest that 25‐HC may have a multipronged mechanism for depleting membrane cholesterol and hence defence against the virus. It is interesting to note that total 25‐HC (sum of biologically active nonesterified 25‐HC and its inactive esterified form) is elevated in patients suffering mild SARS‐CoV‐2 [28], suggesting the successful defence against the virus by 25‐HC may be via its enhanced biosynthesis.

### Key issues to resolve

Despite the availability of vaccines against SARS‐CoV‐2, which are being offered to citizens in rich countries of the developed world, it is questionable whether people in the developing world will be availed such a ‘luxury’. It is also unknown at present how effective the vaccines will be over time. Hence, alternative low‐cost strategies still require exploration. Once such alternative is treatment with the BCG vaccine, which is known to activate the TLR4 [57] and will presumably enhance the expression of IFN [58], so should theoretically enhance *CH25H* expression and the biosynthesis 25‐HC, leading to protection against SARS‐CoV‐2.

#### 7α,25‐dihydroxycholesterol (7α,25‐diHC), 7β,25‐dihydroxycholesterol (7β,25‐diHC) and 25‐hydroxy‐7‐oxocholesterol (25H,7O‐C)

7α,25‐diHC is the major metabolic product of 25‐HC formed in a reaction catalysed by CYP7B1 (EC:1.14.14.29) [59]. It can also be formed from 7α‐hydroxycholesterol (7α‐HC) in a reaction catalysed by CYP3A4 (EC:1.14.14) in human and CYP3A11 (EC:1.14.14) in mouse [60]. 7α,25‐diHC does not show antiviral activity [29], and neither has it been shown to have an effect on SREBP‐2 processing or LXR activation. However, 7α,25‐diHC is a ligand towards the GPCR Epstein–Barr virus‐induced gene 2 (EBI2 or GPR 183) [22, 24] and acts as a chemoattractant to B and T cells expressing the receptor. Hence, in contrast to 25‐HC, which can be regarded as anti‐inflammatory [21, 45], 7α,25‐diHC is a proinflammatory lipokine. Further metabolism of 7α,25‐diHC leads to 7α,25‐dihydroxycholest‐4‐en‐3‐one (7α,25‐diHCO) catalysed by the enzyme hydroxysteroid dehydrogenase (HSD) 3B7 (EC:1.1.1) and further to 7α,25‐dihydroxy‐3‐oxocholest‐4‐en‐26‐oic acid (7α,25‐diH,3O‐CA), probably catalysed by CYP27A1 [61]. Interestingly, 7α,25‐diH,3O‐CA has been found to be of reduced abundance in cerebrospinal fluid (CSF) from patients with Alzheimer’s disease (AD) [62], linking the pathology with proinflammatory 7α,25‐diHC.

Like 7α,25‐diHC, 7β,25‐diHC is also a ligand to GPR183 [22]. However, until recently pathways for the formation of 7β,25‐diHC were unknown [10, 48, 63, 64]. Low levels of both 7β‐hydroxycholesterol (7β‐HC) and 7‐oxocholesterol (7‐OC) are always present in analysis of cholesterol‐derived oxysterols from biological samples [65]. However, the fact that both molecules can be formed from cholesterol via *ex vivo* autoxidation reactions makes interpretation regarding their formation difficult, as similar reactions can also occur endogenously [11]. Convincing evidence for the endogenous nature of 7β‐hydroxy and 7‐oxo metabolites of cholesterol has come from analysis of plasma from people with Niemann–Pick type C disease [47, 48, 66, 67, 68, 69, 70]. Jiang *et al*. provided data showing elevation of 7‐OC, and also of cholestane‐3β,5α,6β‐triol, in plasma from Niemann–Pick type C patients [68]. While more recently, we found elevated levels of both these two cholesterol derivatives and also 7β‐HC in plasma of patients suffering from both Niemann–Pick type C and type B disease [48]. How can we be sure that these are endogenous molecules, not artefacts generated by *ex vivo* autoxidation of cholesterol? Strong evidence for their endogenous nature would be downstream enzymatic products also evident in plasma or urine from Niemann–Pick patients. In fact, Alvelius *et al*. found unusual 7‐oxo‐ and 7β‐hydroxy bile acids in serum and urine from a Niemann–Pick type C patient in 2001 [66], and these identifications were confirmed by Maekawa *et al*. [71], and by Clayton and colleagues, who also identified a bile acid derived from cholestane‐3β,5α,6β‐triol [70]. Further evidence for the *in vivo* nature of 7β‐HC and 7‐OC was the discovery of an entire metabolic pathway from these molecules to 3β,7β‐dihydroxychol‐5‐enoic and 3β‐hydroxy‐7‐oxochol‐5‐enoic acids [48]. In patients with Niemann–Pick disease, it is likely that 7β‐HC and 7‐OC are derived by free radical oxidation of cholesterol [48, 66]. Once formed, 7‐OC and 7β‐HC are interconvertible through the HSD11B enzymes [72, 73]. HSD11B1 (EC:1.1.1) is the 7‐OC reductase, and HSD11B2 (EC:1.1.1), the 7β‐HC dehydrogenase. Both 7β‐HC and 7‐OC are substrates for CH25H, giving 7β,25‐diHC and 25H,7O‐C, respectively, and the two products can be interconverted by HSD11B enzymes [63]. Importantly, 7β,25‐diHC will act as a chemoattractant and activator of GPR183 but 25H,7O‐C will not [22, 63].

However, 25H,7O‐C itself is a biologically active molecule, binding and activating the GPCR protein Smo [64], a member of the Frizzled class of GPCRs. Smo plays a part in the Hh signalling pathway, its activation leading to Hh signalling through Gli (glioma‐associated oncogene homolog) transcription factors. The Hh pathway is essential for proper cell differentiation, and defects in the pathway lead to dysmorphology and cancer. Other key proteins in the Hh pathway are patched‐1 (Ptch1), a sterol transport protein structurally related to NPC1 [74], and the Hh ligand, for example Sonic hedgehog (SHH) post‐translationally modified with cholesterol [75]. Like Niemann–Pick disease, Smith–Lemli–Opitz syndrome (SLOS) is an autosomal recessive monogenetic disorder presenting with elevated 7β‐HC and 7‐OC in plasma and tissues [64, 76, 77]. SLOS also presents with dysmorphology and phenocopies defective Hh signalling [78]. 25H,7O‐C is present at elevated levels in plasma from SLOS patients [64], perhaps acting as a modulator of Smo in competition with other sterol activators. 7β,25‐diHC will also activate Smo and is also found in plasma from SLOS patients [64]. It is likely that the mechanisms behind the biosynthesis of 7β,25‐diHC and 25H,7O‐C in Niemann–Pick disease and SLOS are different. In SLOS, there is a deficiency in 7‐dehydrocholesterol reductase (DHCR7, EC:1.3.1.21), one of the final enzymes in the cholesterol biosynthesis pathways [79], and the consequence of this is a build‐up in 7‐dehydrocholesterol (7‐DHC). Like cholesterol, 7‐DHC is a substrate for CYP7A1 (EC:1.14.14); however, the enzyme products are different, in that the product of CYP7A1 oxidation of 7‐DHC is 7‐OC rather than 7α‐HC, which is formed from cholesterol [76, 80]. Hence, elevated levels of 7‐OC in SLOS are a likely consequence of CYP7A1 oxidation of 7‐DHC. As discussed above, 7‐OC can be reduced to 7β‐HC by HSD11B1 and both can be oxidised to give a 25‐hydroxy product, that is 25H,7O‐C and 7β,25‐diHC, respectively. We have proposed pathways by which 25H,7O‐C and 7β,25‐diHC can be metabolised further to 3β,7β,25‐trihydroxycholest‐5‐enoic acid and ultimately the C_24_ bile acid 3β,7β‐dihydroxychol‐5‐enoic acid [64].

### Key issues to resolve and new ideas

GPR183 has been shown to direct the movement of activated B cells expressing this receptor to outer follicle regions of secondary lymphoid organs as required for mounting a normal B‐cell response to immune challenge. 7α,25‐diHC, 7β,25‐diHC and also 7α,(25R)26‐dihydroxycholesterol (7α,26‐diHC) all act as chemoattractants to B and T cells expressing GPR183. However, the gradient of 7α/β,25‐diHC or 7α,26‐diHC has yet to be measured across lymph nodes to add further evidence to the involvement of these oxysterols in the immune response. One attractive concept is that high levels of 7α,25‐diHC in the lymph node outer follicle attract B cells to mount the inflammatory response, while 7α,26‐diHC, derived from the circulation, reverses the motion, thereby switching off the immune response. Measurements of these oxysterols in tissue should now be possible with the advent of oxysterol mass spectrometry imaging (MSI) [81]. With respect to Hh signalling, Smo and oxysterols, it is unclear how *in vitro* activity of oxysterols translates to the situation *in vivo*, as besides oxysterols, cholesterol will also bind to and activate Smo [75]. If cholesterol rather than oxysterols is the true regulator of the Hh signal, the question is how can such an abundant sterol have signalling functions? Perhaps the answer lies in measuring cholesterol and oxysterol levels in primary cilia, the locality of Smo during the signalling event. Such experiments should now be possible with the advent of sterol‐MSI and will answer the question of whether oxysterols and/or cholesterol dictate Hh signalling [81].

## 24‐hydroxycholesterol (24‐HC)

There are two isomers of 24‐HC, 24S‐HC and 24R‐HC. The 24S‐HC epimer is dominant in man and mouse with 24R‐HC normally constituting of only about 10% of the total in the circulation [82]. 24S‐HC, or cerebrosterol, is as the name suggests mostly synthesised in brain [36, 83]. The enzyme responsible for 24S‐hydroxylation of cholesterol is CYP46A1 (EC:1.14.14.25), which is mostly expressed in neurons [40]. 24S‐HC acts as a transport form of cholesterol providing a route for removal of excess cholesterol from brain by crossing the blood–brain barrier, something that cholesterol itself cannot do [36]. Once extracerebral, 24S‐HC can be sulfated, glucuronidated or converted to bile acids [84, 85, 86]. The 24‐hydroxycholesterol 7α‐hydroxylase is CYP39A1 (EC:1.14.14.26), required to synthesise primary bile acids from 24S‐HC, and is mostly expressed in liver [87].

No inborn error of metabolism has been found resulting from a deficiency in CYP46A1 activity, and the *Cyp46a1* ^−/−^ mouse is viable, showing a comparatively mild phenotype with deficiencies in spatial, associative and motor learning, and in hippocampal long‐term potentiation (LTP) [88, 89]. Interestingly, in these mice the defect in cholesterol metabolism in brain is compensated by its reduced biosynthesis, the overall level of cholesterol in brain being unchanged in the *Cyp46a1* ^−/−^ mouse compared with control [90, 91]. 24S‐HC, like 25‐HC, is a ligand to the LXRs [52, 53] and to INSIG [51], and it is also a modulator of the *N*‐methyl‐D‐aspartate receptors (NMDARs) [92] and of Smo [93]. It is perhaps significant that 24S‐HC, via NMDARs, enhances the ability of subthreshold stimuli to induce LTP [92], considering that the absence of 24S‐HC biosynthesis in the *Cyp46a1* ^−/−^ mouse is linked with a defect in LTP [89].

As 24S‐HC is generated almost exclusively by neurons in brain, its concentration in CSF and plasma has been explored as a marker of neurodegeneration [94]. In early stage disease, one might predict an initial rise in 24S‐HC, as neuronal loss leads to enhanced availability of cholesterol, the substrate for CYP46A1, but at later stages a decay in 24S‐HC as ever‐increasing numbers of neurons, and hence CYP46A1 enzymes, is lost from brain. This can make data interpretation challenging unless samples are well‐stratified. This is illustrated below.

In a recent study, Björkhem *et al*. [95] found 24S‐HC to be elevated in CSF from early Parkinson’s disease (PD) patients in comparison with controls. The same investigators had previously found that CSF 24S‐HC levels correlate with PD disease progression [96]. In contrast to the situation in CSF, the level of 24S‐HC in plasma was not found to differ between PD patients and controls [96]. These data suggest that elevated 24S‐HC in CSF is a marker of neurodegeneration. In patients with AD, 24S‐HC is again elevated in CSF, and this is also true of patients with mild cognitive impairment, but as with PD no differences were found in plasma levels of 24S‐HC [97]. Interestingly, 24S‐HC in CSF was found to increase according to *APOE4* (*apolipoprotein E 4*) status, patients with two *APOE4* alleles having the highest 24S‐HC content of CSF [97]. However, in plasma from severely affected AD patients the same investigators found the 24S‐HC to cholesterol ratio to be decreased in AD [98], presumably as a consequence of loss of CYP46A1 expressing neurons. In a separate study, 24S‐HC was found to be increased in plasma of AD patients, but the levels to negatively correlate with the severity of dementia [99]. Clearly, care must be exercised in stratifying patient samples to maximise the mechanistic insight provided by analytical data. Note, in these studies total 24S‐HC was measured, that is the sum of esterified and nonesterified 24S‐HC.

Levels of 24S‐HC have also been measured in plasma of patients with Huntington’s disease (HD), and concentrations found to vary according to disease severity. Leoni *et al*. [100] measured 24S‐HC in a major study of 150 samples and found that 24S‐HC was elevated in an early progression HD group compared with controls, but reduced compared with controls in a latter progression HD group. These data were at variance with an earlier study performed by Leoni *et al*. who found 24S‐HC to be reduced in HD plasma at all disease states [101].

The CSF and plasma measurements discussed above were all for total 24‐HC, which constitutes the sum of nonesterified and esterified 24‐HC. Usually, the nonesterified, biologically active molecules constitute only about 20% of the total [34]. In the circulation, oxysterols are esterified with fatty acids in a reaction catalysed by the enzyme lecithin–cholesterol acyltransferase (LCAT, EC:2.3.1.43) present in HDL particles. CSF lipoproteins tend to be small and spherical (≈ 10–20 nm)‐like plasma HDL [102], and human CSF contains LCAT at levels corresponding to ∼ 2.5% that of plasma LCAT [103]; however, the very minor levels of nonesterified 24‐HC (0.05 ng·mL^−1^ cf. 1.5 ng·mL^−1^esterified) in CSF indicate that this is sufficient to esterify most of the nonesterified 24S‐HC that is present [62, 104].

### Major unresolved questions

In combination, the data presented above lead to the conclusion that metabolism of cholesterol to 24S‐HC is essential for brain health. However, is 24S‐HC *per se* an essential oxysterol? Evidence from studies on the *Cyp46a1^−/−^* mouse suggests that it is the flow through the cholesterol biosynthesis pathway that is essential rather than 24S‐HC itself [88, 89]. However, 24S‐HC is a modulator of the NMDARs, and a ligand to INSIG, LXRs and Smo, at least *in vitro*, and it is difficult to define its exact importance in activating these pathways as multiple other oxysterols (and sterols) have similar effects on these receptor proteins. A second important question is how good is 24S‐HC as a marker of neurodegeneration? From the studies mentioned above, it is very important to have well‐stratified groups to see a statistical effect. Is this of diagnostic value? The jury is still out.

## 24S,25‐epoxycholesterol (24S,25‐EC)

24S,25‐EC is one of the most efficacious endogenous LXR ligands [52, 53]. It is an unusual oxysterol in that the mechanism of its formation involves cholesterol precursors [105, 106]. There are two likely pathways: (a) 24S,25‐EC may be synthesised in parallel to the Bloch pathway of cholesterol biosynthesis but with squalene epoxidase (SQLE, also named squalene monooxygenase, EC:1.14.14.17) introducing two oxygen atoms, one to give a 2,3S‐epoxide and a second to give a 22S,23‐epoxide, to the squalene skeleton rather than just one to give the 2,3S‐epoxide. The two branches then proceed in parallel to give 24S,25‐EC and cholesterol, respectively, the only difference being that 24‐dehydrocholesterol reductase (DHCR24, EC:1.3.1.72) is absent from the pathway to generate 24S,25‐EC [6, 105, 107]. Lanosterol synthase (LSS, EC:5.4.99.7) is the enzyme that will cyclase both the squalene mono‐ and di‐epoxides, and its reduced activity will encourage di‐epoxide formation and ultimately that of 24S,25‐EC. (b) The alternative pathway to 24S,25‐EC is via CYP46A1 catalysed oxidation of desmosterol [106].

24S,25‐EC is seldom characterised in biological samples [33, 65], and this is a consequence of its comparatively low abundance and the labile nature of the 24S,25‐epoxy group. However, it has been analysed in studies, which do not include an acid or base hydrolysis step [15, 16, 17, 81, 93, 108, 109, 110]. In comparison with other oxysterols, 24S,25‐EC appears to be particularly prevalent in brain during development, perhaps a consequence of a high rate of cholesterol biosynthesis [16, 17, 109, 110, 111]. 24S,25‐EC acts as a ligand towards LXRs [52, 53], and Theofilopoulos *et al*. have generated compelling evidence that 24S,25‐EC, acting through LXRs, promotes midbrain dopaminergic neurogenesis [16, 17]. Besides acting as an LXR ligand, 24S,25‐EC will also bind to INSIG and repress cholesterol synthesis [51]. A more recently uncovered activity of 24S,25‐EC is as a ligand to Smo and activator of the Hh signalling pathway [15, 93, 112]. Cilia are protuberances on the outside of cells, which are required for Smo to transduce Hh signals. Smo accumulates in cilia, and cilia‐associated sterols promote this accumulation and Hh signalling. In search for sterols, which may activate the Hh pathway, Raleigh *et al*. [15] investigated the oxysterols enriched in cilia isolated from sea urchin. One of the oxysterols found was 24S,25‐EC. 24S,25‐EC was found to bind to the extracellular cysteine‐rich domain (CRD) of Smo and activate Smo in a dose‐dependent manner. Interestingly, 24S,25‐EC also activated Hh signalling through mutant Smo missing the CRD [15]. Molecular docking studies suggested 24S,25‐EC also bound to a cytoplasmic binding pocket and mutation studies indicated that Smo activation by 24S,25‐EC was via both binding sites [15]. Ptch1 is key protein involved in inhibition of the Hh pathway, acting as a sterol pump to deplete membranes of sterols. In an effort to identify sterols linked to Hh signalling, Qi *et al*. purified Ptch1 protein and identified 24S,25‐EC as one of the oxysterols co‐purified with Ptch1 [93]. They found evidence for 24S,25‐EC bound to the 7‐transmembrane region of Smo and to be more effective at activating Hh signalling than other sterols [93]. In combination, the data of Raleigh *et al*. and Qi *et al*. establish 24S,25‐EC as a ligand of Smo that can bind to multiple binding pockets and activate Hh signalling [15, 93]. The biological activities of 24S‐HC and 24S,25‐EC appear to overlap in that both activate LXRs, inhibit cholesterol biosynthesis via INSIG and repression of SREBP‐2 processing, and both are ligands to Smo. 24S‐HC and 24S,25‐EC are abundant in brain, and we speculate that brain biology has built a layer of redundancy in that CYP46A1 expressed in neurons and SQLE expressed in glia can each direct synthesis of the biologically active 24S‐oxidised sterols, that is 24S‐HC and 24S,25‐HC, respectively. Perhaps this explains the comparatively mild phenotype of the *Cyp46a1^−/−^* mouse.

### Key issue

The 24S,25‐EC to cholesterol ratio is comparatively high during brain development [16, 17, 109, 110, 111]. This leads us to speculate that during brain development 24S,25‐EC acts as an *in vivo* ligand to Smo and controls Hh signalling and Hh‐linked development.

## (25R)26‐Hydroxycholesterol (26‐HC)

26‐HC, more commonly known by the nonsystematic name 27‐hydroxycholesterol (27‐HC), is the first intermediate in the ‘acidic’, also known as the ‘alternative’, pathway of bile acid biosynthesis [2, 4, 10]. It is synthesised from cholesterol by CYP27A1 (EC:1.14.15.15) and metabolised further to 3β‐hydroxycholest‐5‐en‐(25R)26‐oic acid (3β‐HCA) or to 7α,(25R)26‐dihydroxycholesterol (7α,26‐diHC) by CYP27A1 and CYP7B1, respectively. 3β‐HCA and 7α,26‐HC are both biologically active, the former as a ligand towards LXRs [18, 113], and the latter as a ligand to GPR183 (see above) [22]. 26‐HC is in its own right an LXR ligand, although a comparatively weak agonist [53]. 26‐HC is also a selective oestrogen receptor modulator (SERM) in that it shows anti‐oestrogenic effects or pro‐oestrogenic effects that are cell type‐specific [114, 115]. Oestrogen receptors (ERs) are expressed in vascular cells and mediate cardioprotective effects of oestrogens. However, Umetani *et al*. [114] have shown that 26‐HC can act as a competitive *antagonist* of ER in the vasculature leading to a loss of oestrogen protection towards vascular disease. ERs are also expressed by breast cancer cells, and there is evidence that 26‐HC acts as a partial *agonist* in these cells [19, 115, 116]. Given that 26‐HC is a direct product of cholesterol metabolism, these findings have implications with respect to breast cancer and hypercholesteraemic women. In fact, Wu *et al*. found that in ER(+) breast cancer (ER(+)BC) patients the 26‐HC content of normal tissue was higher than that from controls, and tumour 26‐HC levels were further elevated [116]. In a study published at almost exactly the same time, Nelson *et al*. [19] reported that in breast tissue CYP27A1 levels correlate with tumour grade. Nelson *et al*. [19] showed that 26‐HC stimulated ER(+)BC proliferation through the ER and invasiveness through LXR. In a later study, Nelson [117] proposed that inhibition of CYP27A1 along with ERα and LXR antagonists could increase the efficacy of treatments towards ER(+)BC.

Surprisingly, in the light of the discussion above, a systematic review and meta‐analysis of prospective studies found a modest but statistically significant inverse association between total cholesterol, more specifically HDL cholesterol, and the risk of breast cancer [118]. In addition, in a study of almost 300 breast cancer cases no association was found between circulating 26‐HC and breast cancer risk [119], and in postmenopausal women, circulating 26‐HC was associated with a lower risk of breast cancer [120]. Unfortunately, the authors of these reports did not clarify whether they were measuring the total 26‐HC, that is the sum of the inactive ester and the active nonesterified molecule or just the active nonesterified molecule. The reader is left to guess, but in all probability a reported value of about 200 nm (80 ng·mL^−1^) in plasma refers to the total 26‐HC. As discussed elsewhere, there is a need for clarity in reporting of mass spectrometry data [121].

Like 25‐HC, nonesterified 26‐HC has been suggested to be an antiviral oxysterol [28]. Marcello *et al*., measuring total 26‐HC, found the plasma level of this oxysterol in severely affected COVID‐19 patients to be almost half that in control subjects [28]. Serum levels of cholesterol and its precursors were low in both moderate and severe COVID‐19 cases, possibly explaining reduced levels of 26‐HC. Notably, levels of antiviral 25‐HC were also reduced in severe COVID‐19 cases, although the reduction was not great (8.52 ± 2.58 ng·mL^−1^ in controls cf. 7.64 ± 2.49 ng·mL^−1^ in severe cases). Importantly, the reader should be reminded that total sterols were being measured not the nonesterified bioactive molecules.

Perhaps the reduced availability of cholesterol to cells is a key aspect in the pathophysiology of COVID‐19 leading to reduced biosynthesis of antiviral 25‐HC and 26‐HC. However, based on studies suggesting 25‐HC is antiviral through reducing cholesterol availability to membranes [29] it might be expected that reduced serum cholesterol would be beneficial in protection against COVID‐19.

### Key issue

Are total oxysterol levels a good surrogate measure for concentrations of the nonesterified bioactive molecules? As LCAT is abundant in HDL particles and will esterify sterols, it not an unreasonable assumption that the levels of esterified oxysterols *are* reflective of bioactive nonesterified oxysterols exported from cells. However, care should be taken when relating concentrations of the total oxysterol measured in plasma to that required for an *in vitro* or *in vivo* biological activity.

#### Cholestenoic acids: 3β‐HCA, 3β,7α‐dihydroxycholest‐5‐en‐(25R)26‐oic (3β,7α‐diHCA), 3β,7β‐dihydroxycholest‐5‐en‐(25R)26‐oic (3β,7β‐diHCA) and 3β‐hydroxy‐7‐oxocholest‐5‐en‐(25R)26‐oic (3βH,7O‐CA) acids

3β‐HCA and 3β,7α‐diHCA are intermediates in the acidic pathway of bile acid biosynthesis [4, 10], and both these molecules, and the downstream metabolite 7α‐hydroxy‐3‐oxocholest‐4‐en‐(25R)26‐oic acid (7αH,3O‐CA), are present in CSF and/or brain [41, 62, 81]. Meaney *et al*. [122] have shown that 7αH,3O‐CA provides a metabolic export route for 26‐HC from brain, which itself is imported to brain, and is the first metabolite in the acidic pathway of bile acid biosynthesis [4, 10]. Both 3β‐HCA and 3β,7α‐diHCA are LXR ligands, as are 3β,7β‐diHCA and 3βH,7O‐CA, but not 7αH,3O‐CA [18, 113]. Interestingly, Theofilopoulos *et al*. showed that in the developing brain 3β,7α‐diHCA promoted motor neuron survival in an LXR‐dependent manner, 3βH,7O‐CA promoted maturation of precursors into motor neurons, while 3β‐HCA was toxic, showing that cholestenoic acids dictate the balance between life and death of motor neurons [18]. In a more recent study, Abdel‐Khalik *et al*. [64] have shown that 3β,7β‐diHCA and 3βH,7O‐CA activate Hh signalling by binding to Smo, highlighting a further potential role for cholestenoic acids in development.

### New ideas

In the light of the biological activity of 26‐HC, 7α,26‐diHC and of the cholestenoic acids, we speculate that the acidic pathway evolved as more than a pathway of bile acid biosynthesis. The widespread expression of CYP27A1 contrasts to that of liver‐specific CYP7A1, the first enzyme in the neutral pathway of bile acid biosynthesis [4, 10], and it may also be significant that CYP27A1 is an inner mitochondrial enzyme, in contrast to most other CYPs involved in cholesterol metabolism, which are localised to the endoplasmic reticulum. We suggest that the acidic pathway initially evolved to generate biologically active signalling molecules and its development within hepatocytes provided the bonus of bile acid formation to remove excess cholesterol and to aid absorption in the intestine of dietary lipids. This hypothesis is supported by the activity of the acidic pathway during embryonic development [18, 109] and data that suggest that in infants the acidic pathway is more important than the neutral pathway of bile acid biosynthesis [123]. Kakiyama *et al*. [124] have proposed a somewhat similar evolutionary role for bile acid biosynthesis. They suggest that the acidic pathway evolved as a mechanism to remove excess cholesterol from the inner mitochondrial leaflet by CYP27A1 metabolism to 26‐HC, with further metabolism regulated by endoplasmic reticulum‐resident CYP7B1. They then proposed that an inability of the acidic pathway to increase the synthesis of bile acids without generating toxic intermediates leads to evolution of the neutral pathway starting with CYP7A1 and generating less‐toxic intermediates [124]. The two hypothesises differ in that Kakiyama and Pandak focus on toxic intermediates and a requirement for a different and neutral pathway [124], while we focus more on the positive effects of intermediates of the acidic pathway.

## Cholestan‐5,6‐epoxide (5,6‐epoxycholesterol, 5,6‐EC) and cholestane‐3β,5α,6β‐triol

**Fig. 3:**
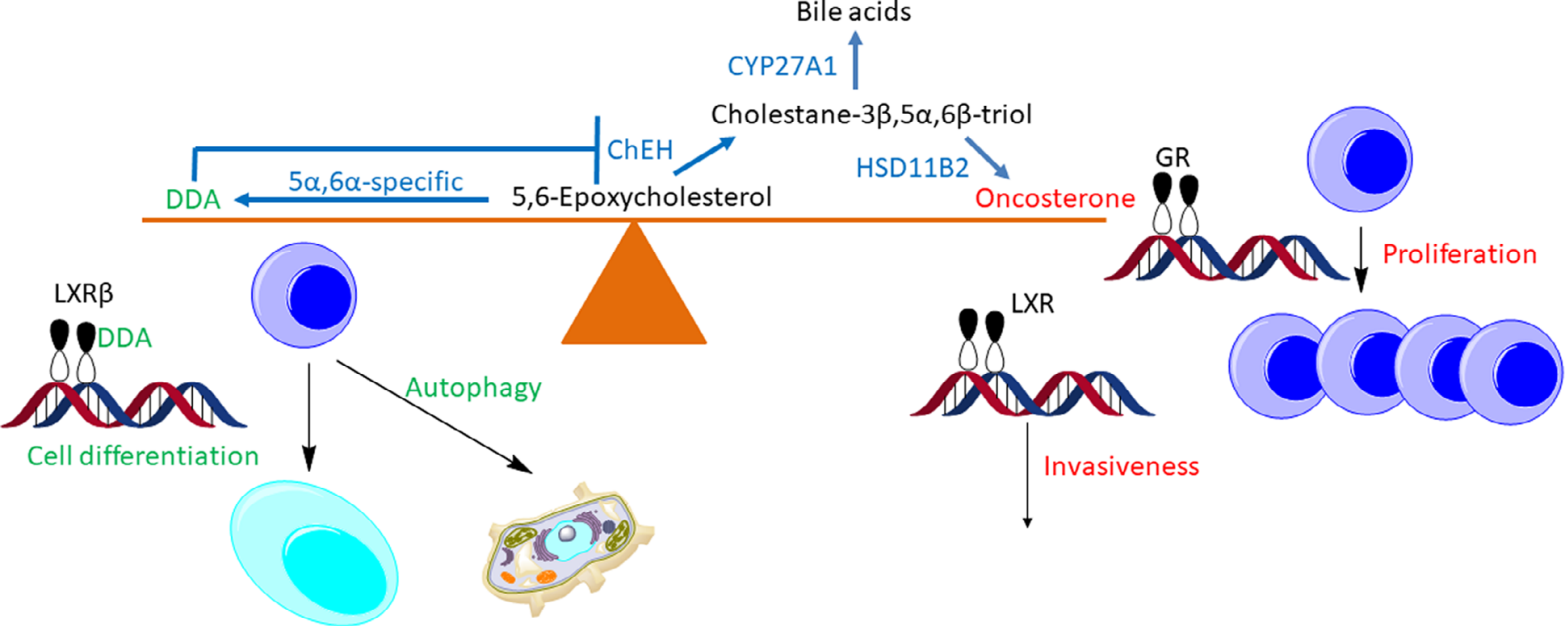
5,6‐EC at the fulcrum of protection against and the proliferation of cancer.

There are two isomers of 5,6‐EC with either 5α or 5β stereochemistry. Both are formed by free radical oxidation of cholesterol [11]. Both isomers can be hydrolysed by the enzyme cholesterol epoxide hydrolase (ChEH, EC:3.3.2.11) to cholestane‐3β,5α,6β‐triol. ChEH is an unusual enzyme in that it made up of two subunits, each of which is an enzymes in its own right, and part of the cholesterol biosynthesis pathway, that is DHCR7 and 3β‐hydroxysterol‐Δ^8^‐Δ^7^‐isomerase (D8D7I, EC:5.3.3.5) [125]. As mentioned above, cholestane‐3β,5α,6β‐triol is elevated in the circulation people with Niemann–Pick type C and type B disease and also those with lysosomal acid lipase (LIPA, EC:3.1.1.13) deficiency, known as Wolman disease when there is a complete absence of the enzyme [48, 68, 126]. The origin of cholestane‐3β,5α,6β‐triol is likely to be via hydrolysis of 5,6‐EC, which is also elevated in Niemann–Pick and in Wolman diseases [48]. Cholestane‐3β,5α,6β‐triol can be metabolised via multiple reactions to bile acids [48, 69, 70], or alternatively oxidised by HSD11B2 to 3β,5α‐dihydroxycholestan‐6‐one (oncosterone, 3β,5α‐diHC‐6O). As the trivial name oncosterone implies, 3β,5α‐diHC‐6O is a tumour promoter (Fig. 3) [127].

Oncosterone has been shown to promote proliferation of mouse and human ER(+)BC and triple‐negative breast cancer cell lines, and growth of breast cancer tumours *in vivo* [127]. The proliferative effects of oncosterone are through its activation of the glucocorticoid receptor (GR) [127]. Interestingly, corticosteroid ligands to GR do not share the proliferative activities of oncosterone [9]. Oncosterone also acts as a ligand to the LXR receptors, and it has been suggested that the pro‐invasive effects of oncosterone are mediated by LXR [127].

An alternative route for metabolism of 5α,6‐EC, but not 5β,6‐EC, is enzymatic conjugation with an amine nucleophile. Two such nucleophiles showed to react with 5α,6‐EC in chemically catalysed reactions are histamine and spermidine to give dendrogenin A (DDA) and dendrogenin B (DDB), respectively. Both of these metabolites have now been identified in mammalian systems [128]. In contrast to oncosterone, DDA is oncosuppressive. Importantly, DDA is not detected in cancer cell lines, and its level in breast tissue is decreased during oncogenesis [129]. The oncosuppressive effects of DDA are at least part through activation of LXRβ, inducing autophagy and cell differentiation [130], while a second pathway is through inhibition of ChEH and follow‐on inhibition of biosynthesis of oncosterone.

### A Role in therapeutic development

The studies discussed above regarding metabolism of 5,6‐EC indicate three potential targets for pharmaceutical intervention:

1. The balance between cholestane‐3β,5α,6β‐triol and oncosterone is dependent on the enzymes HSD11B2 and HSD11B1. Inhibition of HSD11B2 will reduce the formation of oncosterone.
2. ChEH generates cholestane‐3β,5α,6β‐triol from 5,6‐EC. Inhibition of ChEH should reduce the formation of cholestane‐3β,5α,6β‐triol and consequently that of oncosterone. Likewise, inhibition of ChEH should shunt 5α,6‐EC towards the biosynthesis of DDA that is oncosuppressive through LXRβ.
3. A quite different idea is the upregulation of CYP27A1. Assuming from the studies of Le Cornet and coworkers that there is no correlation between circulating 26‐HC and breast cancer risk [119, 120], and in direct contrast to the suggestion of Nelson to inhibit CYP27A1 [117], enhancing CYP27A1 expression or activity should drive cholestane‐3β,5α,6β‐triol into the bile acid biosynthesis pathway and away from metabolism by HSD11B2 to oncosterone. These ideas are yet to be tested.

## 22R‐hydroxycholesterol (22R‐HC)

Cholesterol is converted to 22R‐HC by CYP11A1 (EC:1.14.15.6). Like CYP27A1, CYP11A1 is a resident of the innermitochondrial membrane. CYP11A1 can then convert 22R‐HC to 20R,22R‐dihydroxycholesterol (20R,22R‐diHC) and ultimately the C_21_ steroid, pregnenolone. These reactions may or may not proceed with the release of the oxysterol intermediates [131, 132]. Like other side‐chain oxysterols, 22R‐HC is a ligand to LXRs and an inhibitor of SREBP‐2 processing to its active form [51, 52]. The metabolism of 20R‐HC and 20R,22R‐diHC to bile acids rather than steroids is another story yet to be told.

## Cholesterol precursors and their oxysterol derivatives

The hypothesis that the acidic pathway evolved to generate biologically active intermediates can be extended to include the Kandutsch–Russell and Bloch pathways of sterol biosynthesis. Lanosterol, the first sterol, has been shown to stimulate INSIG‐mediated degradation of HMGCoA reductase (HMGCR, EC:1.1.1.34), the enzyme catalysing the rate‐determining step of cholesterol synthesis, and remarkably, 26‐hydroxylanosterol, also called 27‐hydroxylanosterol, is 10 times more potent [133]. Other sterols in the Kandutsch–Russell and Bloch pathways with 4,4‐dimethyl groups were shown to similarly accelerate the degradation of HMGCR, as does 25‐HC [133, 134]. Lanosterol does not repress the SREBP‐2 activation of cholesterol biosynthesis, but in contrast to initial data [133], other 4,4‐dimethyl sterols are reported to inhibit SREBP‐2 activation [134]. At the other end of the cholesterol biosynthesis pathway from lanosterol, desmosterol inhibits the processing of SREBP‐2 and acts as a ligand towards LXRs [135]. As discussed above, desmosterol acts as a substrate for CYP46A1 generating biologically active 24S,25‐EC [106], which can also be formed in a shunt of the Bloch pathway [105]. 7‐DHC, the final member of the Kandutsch–Russell pathway, is also a source of bioactive metabolites, and these may be formed enzymatically, or via free radical reactions, 7‐DHC being particularly susceptible to nonenzymatic oxidation. For example, 3β,5α‐dihydroxycholest‐7‐en‐6‐one, formed by hydrolysis and oxidation of 7‐dehydrocholesterol‐5α,6‐epoxide, is a Hh pathway antagonist binding to Smo at a site distinct from the CRD and the cyclopamine pocket [136]. As discussed earlier, 7‐DHC can be converted to 7‐OC by CYP7A1 [80], and this opens a route to 7β‐HC and multiple biologically active oxysterols [22, 64, 137].

## Conclusions

Oxysterols and other intermediates in the bile acid biosynthesis pathways have interested sterol chemists for decades [13]. It appears that while the neutral pathway of bile acid biosynthesis generates intermediates with comparatively little biological activity, the reverse appears to be the case with the acidic pathway. The prominence of the acidic pathway in early life highlights the importance of its intermediates, and as suggested above, it is tempting to speculate that the acidic pathway evolved to generate and regulate biologically active molecules, and its utilisation by hepatocytes provided an added bonus of generating bile acids.

**Fig. 4:**
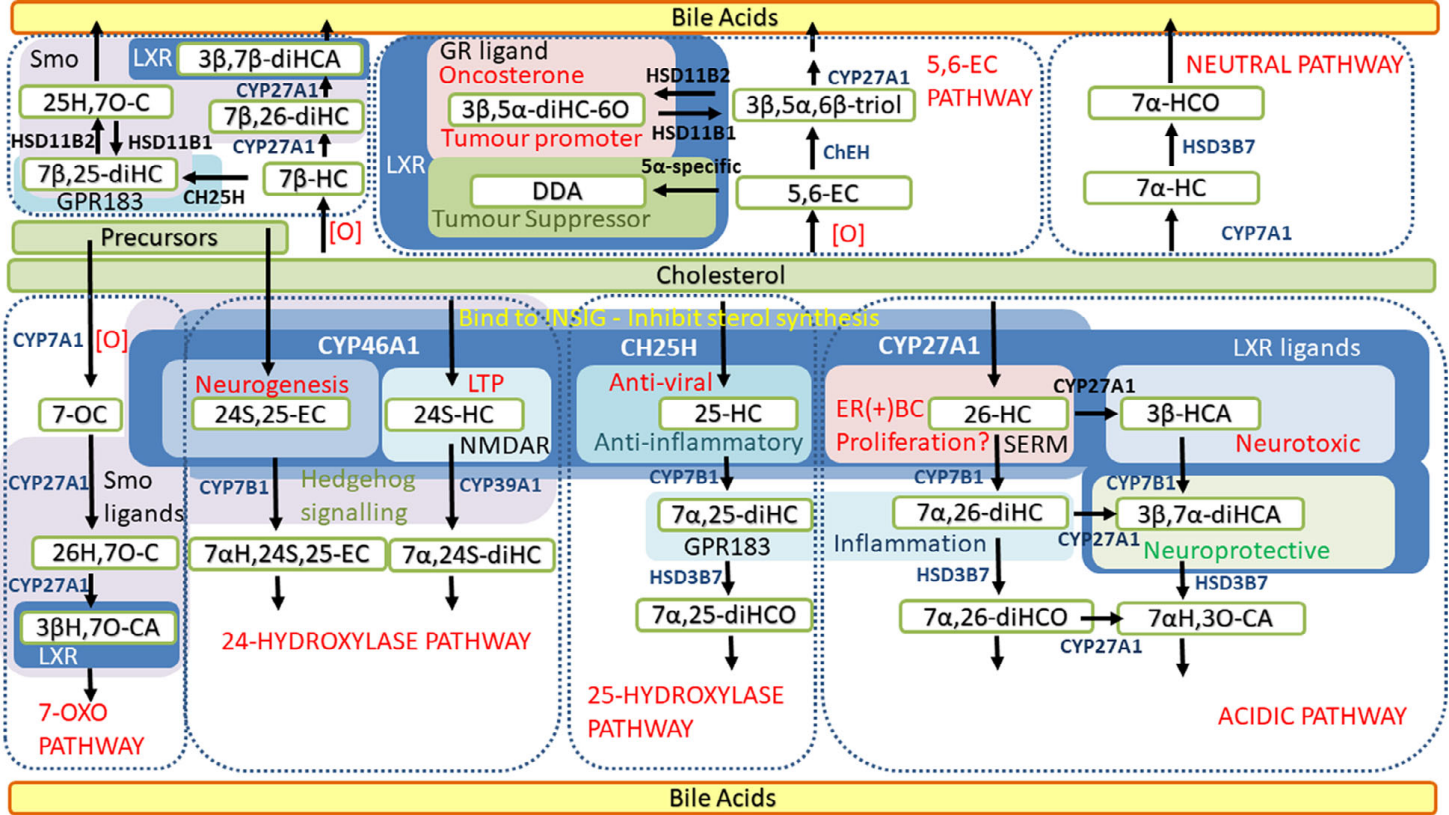
The acidic pathway of bile acid biosynthesis generates bioactive intermediates. Key enzymes in this pathway, CYP27A1 and CYP7B1, can be lent to different bile acid biosynthesis pathways to similarly generate and regulate other lipokines.

Besides the acidic pathway, other pathways to bile acids, and also to steroid hormones, generate bioactive intermediates. In which case, progression towards bile acids may also be a way of regulating lipokine biosynthesis and metabolism in specific cell types and tissues, for example CYP46A1 is expressed in brain; CH25H in activated immune cells; and CYP11A1 in sex organs and the adrenal gland. In contrast to these three enzymes, CYP27A1, initiating the acidic pathway, and CYP7B1 acting as the 7α‐hydroxylase in this pathway are rather ubiquitously expressed and can be ‘lent’ to the different bile acid biosynthesis pathway to help regulate the formation/metabolism of the active intermediates (Fig. 4). Of course, an ultimate function of bile acid formation is also to remove excess cholesterol.

Bile acids can also be synthesised from oxysterols generated by nonenzymatic reactions. Incredibly, intermediates in these pathways also have biological activity. While 7‐OC and 7β‐HC can also be formed enzymatically, an enzyme to generate 5,6‐EC has yet to be isolated. Is there one, or has the pathway from 5,6‐EC evolved exclusively to remove products of cholesterol oxidation formed by reactive oxygen species?

The multiple pathways to bile acids provide redundancy in the biological system with many intermediates sharing similar activities, and this is probably the reason why most of the inborn errors of bile acid biosynthesis are not fatal, and the equivalent knockout mice are viable. However, these inborn errors do lead to disease indicating the imperfection of the back‐up system.

The huge range of bile acid intermediates, the crossover of pathways, sharing of enzymes, shuttling of oxysterols between different organelles, cell types and tissues make this a fascinating field to work in, with still many important discoveries to be made.

## Conflict of interest

WJG and YW are listed as inventors on the patent ‘Kit and method for quantitative detection of steroids’ US9851368B2. WJG and YW are listed as inventors on the patent application ‘Diagnostic methods and kits’ WO2017037465A1. WJG, EY and YW are shareholders in CholesteniX Ltd.

## Author contributions

WJG and YW conceived the paper. EY provided valuable insight regarding oxysterol analysis. All authors contributed to the writing of the paper, and reviewed and edited the final version.
